# Surgical care for the direct and indirect victims of violence in the eastern Democratic Republic of Congo

**DOI:** 10.1186/1752-1505-4-6

**Published:** 2010-04-14

**Authors:** Kathryn Chu, Philippe Havet, Nathan Ford, Miguel Trelles

**Affiliations:** 1 Médecins sans Frontières, 49 Jorrisen St, Braamfontein 2017, Johannesburg, South Africa; 2 Departments of Surgery and International Health, Johns Hopkins University, Baltimore, MD, USA; 3 Médecins sans Frontières, Masisi, Democratic Republic of Congo; 4 Faculty of Health Sciences, Simon Fraser University, Vancouver, Canada; 5 Médecins sans Frontières, rue Dupré 94, 1090 Brussels, Belgium

## Abstract

**Background:**

The provision of surgical assistance in conflict is often associated with care for victims of violence. However, there is an increasing appreciation that surgical care is needed for non-traumatic morbidities. In this paper we report on surgical interventions carried out by Médecins sans Frontières in Masisi, North Kivu, Democratic Republic of Congo to contribute to the scarce evidence base on surgical needs in conflict.

**Methods:**

We analysed data on all surgical interventions done at Masisi district hospital between September 2007 and December 2009. Types of interventions are described, and logistic regression used to model associations with violence-related injury.

**Results:**

2869 operations were performed on 2441 patients. Obstetric emergencies accounted for over half (675, 57%) of all surgical pathology and infections for another quarter (160, 14%). Trauma-related injuries accounted for only one quarter (681, 24%) of all interventions; among these, 363 (13%) were violence-related. Male gender (adjusted odds ratio (AOR) = 20.0, p < 0.001), military status (AOR = 4.1, p < 0.001), and age less than 20 years (AOR = 2.1, p < 0.001) were associated with violence-related injury. Immediate peri-operative mortality was 0.2%.

**Conclusions:**

In this study, most surgical interventions were unrelated to violent trauma and rather reflected the general surgical needs of a low-income tropical country. Programs in conflict zones in low-income countries need to be prepared to treat both the war-wounded and non-trauma related life-threatening surgical needs of the general population. Given the limited surgical workforce in these areas, training of local staff and task shifting is recommended to support broad availability of essential surgical care. Further studies into the surgical needs of the population are warranted, including population-based surveys, to improve program planning and resource allocation and the effectiveness of the humanitarian response.

## Background

The provision of surgical assistance in conflict is often associated with care for victims of violence. However, there is an increasing appreciation that surgical care is needed for non-traumatic morbidities [1]. Armed conflict often occurs in low-income countries where fragile health care systems are rapidly overwhelmed during periods of violence and associated population displacement. In such situations, the population becomes even more vulnerable to threats such as poor hygiene, malnutrition, infectious diseases, rape, and poor antenatal care [2]. Infections and obstetric emergencies in particular contribute to substantial mortality in these settings and surgical interventions can make an important contribution to reducing death and disability [3].

An accurate understanding of the surgical needs of populations in conflict is therefore important for program planning and resource allocation. Program audits for Médecins sans Frontières (MSF) operations during 2008 found that only 30% of surgical interventions were due to violence-related injuries; the majority of interventions were for obstetric emergencies and accidental trauma [1]. In this paper we report on surgical interventions carried out by MSF in a zone of active conflict in Masisi, North Kivu, Democratic Republic of Congo, and describe risk factors for violence-related injury.

## Methods

### Setting

The Democratic Republic of Congo (DRC) is one of the poorest countries in the world with a per capita GDP of $300 [4]. North Kivu, located in Eastern DRC, has been afflicted by conflict since the Rwandan genocide in 1994 which resulted in millions of refugees fleeing into the region. Its close proximity to Uganda and Rwanda also make it vulnerable to incursions by armed groups in these countries. There are various government and rebel factions, but the main conflict is between the DRC military and two militia groups, the Hutu-based Democratic Forces for the Liberation of Rwanda and (the now defunct) National Congress for the Defense of the People (CNDP).

MSF has been in North Kivu since 1992, providing medical services to the displaced and host populations. The estimated 1.4 million internally displaced persons who live in North Kivu make up almost half (47%) of the total population [5]. Life expectancy is 46 years [6] and leading causes of death are malaria, diarrhea, respiratory infections, tuberculosis, and neonatal deaths [7]. The terrain is mountainous and poor roads mean the area is only accessible by all terrain vehicles and motorbikes for most of the year.

On August 27, 2007, the CNDP attacked the village of Masisi, located 85 km from Goma, the capital of North Kivu. Heavy fighting lasted for four days, with small skirmishes continuing for several weeks. Tens of thousands of new IDPs were estimated to have fled the area and while the number of casualties was not reported, similar waves of violence in the DRC are known to have resulted in high civilian mortality [8]. In response, MSF established surgical services in Masisi district hospital to treat the war wounded.

Masisi district has 30 primary health clinics, although many are non-functional due to lack of human resources or essential supplies. The catchment population is difficult to establish because of continuous population displacement but is estimated to be around 306,000 people. Masisi district hospital was established in the late 1960s as the referral hospital for the district. After the August 2007 attacks in Masisi, the hospital was practically abandoned as staff and patients fled to safer areas. Continued fighting has occurred over the past two years with waves of increased violence. MSF began the provision of emergency surgical services in September 2007 by renovating the operating theatre, sterilization unit, and providing surgeons and anesthesiologists. Surgical care is provided by an MSF surgeon with the assistance of Congolese government doctors, while anesthestic services are provided by an MSF anesthesiologist and a Congolese nurse-anesthetist. Over time MSF has expanded support to cover all inpatient services including maternity, pediatrics, and internal medicine, as well as laboratory services and the emergency room. Currently there are 32 surgical beds in the 175-bed hospital. MSF is responsible for all medication and supplies and provides services free of charge to patients.

### Data Sources

For this analysis, we defined surgical interventions as all procedures that required anesthesia and were performed in the operating room. The period of analysis was from September 2007 to December 2009. The following data were prospectively collected using Excel: age, gender, military status, and American Society of Anesthesiology (ASA) physical status classification as well as data on surgical pathology, procedure type, blood transfusions, and operative mortality. Surgical pathology was grouped into the following categories: obstetric emergencies, infection, neoplasm, accidental injury, violence-related injury, and other.

### Statistical analysis

Baseline characteristics were described using medians and interquartile ranges (IQRs) for continuous variables and counts and percentages for categorical data. Associations with violence-related injury were explored using using logistic regression. Variables considered in the analysis included age, gender, military status, ASA classification, and blood transfusions. Factors with a p < 0.1 on univariate analysis were included in a multivariate model. All tests and confidence intervals were considered to be significant at a p ≤ 0.05. All analyses were performed using STATA 10 (College Station, TX, USA).

## Results

From September 2007 to December 2009, 2869 operations were performed on 2441 patients (15% re-interventions). The majority (1855, 76%) were female. Median age was 24 (interquartile range 18-31); 152 (6%) were under 5 years of age. Sixty-one patients (3%) were in the military.

1263 (44%) procedures were performed under spinal anesthesia; 1263 (44%) under general anesthesia without intubation, and only 115 (4%) under general anesthesia with intubation. Immediate peri-operative mortality was 0.2% (20); however, in-hospital mortality was unknown.

### Surgical Pathology

**Figure 1:**
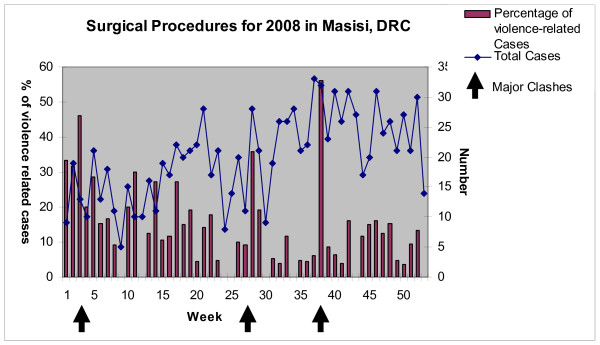
**Surgical procedures for 2008 in Masisi, DRC**.

We found that obstetric emergencies accounted for over half (1463, 51%) of all surgical pathologies, and infections for another quarter (498, 17%). Trauma-related injuries accounted for only one quarter (681, 24%) of all interventions; among these, 363 (13%) were violence-related (Table 1). The proportion of violence-related cases varied from 0-56%, with peaks occurring during major clashes (Figure 1). Gunshot wounds accounted for 94% (341) of violent injuries (Table 2). The most common non violence-related injuries were burns and falls. The most common procedure for trauma was wound debridement while the most common non-trauma-related procedure was Cesarean section (Table 3).

**Table 1:** Types of Surgical Pathology

	N	(%)
Obstetrical Emergencies	1463	(51)
Infections	498	(17)
Violence-related Injuries	363	(13)
Accidental Injuries	318	(11)
Other*	142	(5)
Neoplasms	82	(3)

	2869	(100)

*Congenital, Iatrogenic, Vascular, Other Pathology

**Table 2:** Causes of Violent Injury

	N	(%)
Gunshot Wound	341	(94)
Knife	13	(4)
Torture	5	(1)
Rape	3	(1)
Missile	1	(0)

Total	363	(100)

**Table 3:** Trauma and Non-Trauma Related Interventions

Trauma	N	(%)	Non-Trauma	N	(%)
Wound Debridement	206	(30)	Cesarean section	1304	(60)
Dressing Changes under Sedation	184	(27)	Suturing, I and D, Circumcision	237	(11)
Suturing, Incision and Drainage	126	(19)	Tubal ligation/Dilation and curretage	113	(5)
Fracture Reductions	64	(9)	Dressing Changes under Sedation	112	(5)
Abdominal Surgery/Bowel Resection	21	(3)	Abdominal Surgery*	97	(4)
Amputations	20	(3)	Wound Debridments	94	(4)
Skin Grafts	17	(2)	Minor Surgery**	81	(4)
Other	43	(6)	Other	76	(3)

Total	681	(100)	Total	2188	(100)

*Bowel resections, appendectomies, removal of tumors

**Herniorraphy, Hydrocele repair, hemmorrhoid surgery

### Associations with violence-related injury

We assessed risk factors for violence-related injury using a multivariate model that included sex, age, military status, ASA classification, and the provision of blood transfusions. Among these, the following were found to be statistically significantly associated with violence-related injury: male gender (adjusted odds ratio (AOR) = 19.2, p < 0.001), military status (AOR = 4.1, p < 0.001), and age less than 20 years (AOR = 2.1, p < 0.001) (Table 4).

**Table 4:** Associations with Violence-related Injury

	**Univariate**			**Multivariate**		
	
	**OR**	**95% CI**	**P**	**OR**	**95% CI**	**P**
		
Female	1.0					
Male	20.0	(14.0-28.6)	<0.001	19.2 (13.1-28.1)		<0.001
						
Age ≥ 20 years	1.0					
Age < 20 years	1.4	(1.0-2.0)	0.051	2.1	(1.5-3.0)	<0.001
						
Civillian	1.0					
Military	23.4	(13.5-40.6)	<0.001	4.1	(2.3-7.4)	<0.001
						
ASA 1-2	1.0					
ASA 3-5	2.6	(1.5-4.4)	<0.001	1.5	(0.8-2.8)	0.221
						
No blood transfusion	1.0					
Blood transfusion	2.2	(1.0-5.0)	0.063	2.8	(0.9-8.5)	0.068

ASA, American Society of Anesthesiologists physical status classification system

## Conclusions

This is among the few studies to describe the typology of emergency surgical care in a conflict zone. We found that in this war-ravaged area of Eastern DRC most surgical interventions were unrelated to violent trauma but rather reflected the general surgical needs of a low-income tropical country. Most of the emergency procedures performed were similar to those performed in hospitals in low-income countries not in conflict [9,10]. The prevention of maternal and fetal mortality was the most common reason for emergency surgery in the Masisi program. Like many populations who suffer a general lack of access to primary health care, this population suffered from accidents, infections, and late-stage neoplastic infections.

These findings are consistent with program audits from other conflict zones in resource-limited settings. A retrospective review of surgical services of Médecins Sans Frontières in six conflict-settings in both Africa (Chad, Somalia, South Sudan, Democratic Republic of Congo, and Central African Republic) and South Asia (Pakistan) found that only 22% of surgical interventions were due to violent injury, while obstetric emergencies accounted for almost a third (30%) of interventions and accidental injury and infections another third [1].

Male soldiers younger than 20 years of age were more likely to present with violent trauma. While those patients suffering from violent trauma only accounted for 13% of the surgical cases, they needed special support such as long-term rehabilitation and psychological counseling. Hospitals in war zones should plan for these needs.

Our study demonstrated that while this project had fully trained surgeons and anesthesiologists, most of the procedures performed were basic. This was in part due to the limitations of the equipment and the lack of post-operative intensive care. Also, there was likely to be a selection bias against complex trauma, as patients with severe head or chest trauma likely never made it to the hospital as pre-hospital transport took hours to days. Nevertheless, this finding is important as it indicates that most procedures can be performed by general doctors or non-physician clinicians with surgical skills. For example, it has been shown that the most common surgical intervention, emergency obstetrical care, can with adequate training and supervision be performed safely performed by non-doctors [11-13]. In low-income settings such as Niger, Malawi, and Mozambique, surgical task-shifting has resulted in an increased provision in essential surgical services [14,15]. Similarly, most of these procedures were safely performed with spinal anesthesia and ketamine (general anesthesia without intubation) which are safer types of anesthesia to administer for nurse-anesthetists or anesthesia providers that are informally trained.

The potential for non-surgeons to manage a substantial proportion of surgical needs in resource-limited conflict areas is an important consideration given the lack of local surgeons in resource-limited settings [16] and the danger posed to expatriate surgeons (in particular, the higher risk of kidnapping in certain contexts). In Somalia, where MSF expatriate surgeons are not allowed due to insecurity, all surgical procedures are performed by non-surgeons; operative mortality is <1%. Studies from other settings demonstrate that the training of general doctors with surgical skills and nurse anesthetists is possible, even in a conflict zone [16,17].

This study has certain limitations. The reported numbers of war-wounded were often higher than the number of victims treated at Masisi district hospital, which was the only health care facility providing surgical care in this community. While some likely died prior to arriving at the hospital from severe injuries, others may not have sought care. This study did not measure reasons for service uptake. While all care was free, there may have been other barriers to accessing care including transportation, insecurity, and other family responsibilities. Civilians and soldiers from both sides of the conflict were treated confidentially and respectfully by hospital staff; however, regional and tribal differences between staff and patients may have prevented some patients from seeking care. Special attention to improve access to care for the war-wounded and IDPs is needed.

Collecting data in conflict settings is challenging, but not impossible [18]. Our study was limited by our data collection methods. While our coding system captured broad categories of surgical pathology, it was limited in documenting types of operations. The coding system did not distinguish between some minor surgeries such as herniorraphy, hydrocele repair, and hemorrhoid surgery or wound suturing, incision and drainage of abscesses, and circumcision. Knowing the exact cause of many diseases without radiology or pathology services was also difficult. We did not have long-term follow-up of patients nor did we track surgical site infection. While this study described the burden of essential surgical disease in a conflict zone, it could not determine the burden of elective surgical disease. Even though many patients with elective surgical disease were evaluated at the hospital, this was unlikely representative of all the type of surgical disease in the community. Population based studies are needed to estimate the unmet burden of elective surgical disease.

In conclusion, programs in conflict zones in low-income countries need to be prepared to treat both the war-wounded and non-trauma related life-threatening surgical needs of the general population. While military patients have a greater relative risk of violence-related injuries, civilians still make up the majority of violence-affected cases in terms of absolute numbers. Training of local staff and task-shifting is essential to ensure that surgical services will be provided when conditions become too dangerous for expatriate surgeons to work in the area. Further studies into the surgical needs of the population are warranted, including population-based surveys, to improve program planning and resource allocation and ultimately the effectiveness of the humanitarian response.

## Competing interests

The authors declare that they have no competing interests.

## Authors' contributions

KC, NF, and MT were responsible for the overall concept and design. KC and MT contributed to the data collection and analysis. KC, NF, and MT contributed to intellectual content, and writing of the paper. KC wrote the first draft of the paper. All authors reviewed and approved the final version of the paper.
